# The SUSTech-SYSU dataset for automatically segmenting and classifying corneal ulcers

**DOI:** 10.1038/s41597-020-0360-7

**Published:** 2020-01-20

**Authors:** Lijie Deng, Junyan Lyu, Haixiang Huang, Yuqing Deng, Jin Yuan, Xiaoying Tang

**Affiliations:** 1 Department of Electrical and Electronic Engineering, Southern University of Science and Technology, Shenzhen, China; 2 State Key Laboratory of Ophthalmology, Zhongshan Ophthalmic Center, Sun Yat-sen University, Guangzhou, China

**Keywords:** Corneal diseases, Data processing, Fluorescence imaging

## Abstract

Corneal ulcer is a common ophthalmic symptom. Segmentation algorithms are needed to identify and quantify corneal ulcers from ocular staining images. Developments of such algorithms have been obstructed by a lack of high quality datasets (the ocular staining images and the corresponding gold-standard ulcer segmentation labels), especially for supervised learning based segmentation algorithms. In such context, we prepare a dataset containing 712 ocular staining images and the associated segmentation labels of flaky corneal ulcers. In addition to segmentation labels for flaky corneal ulcers, we also provide each image with three-fold class labels: firstly, each image has a label in terms of its general ulcer pattern; secondly, each image has a label in terms of its specific ulcer pattern; thirdly, each image has a label indicating its ulcer severity degree. This dataset not only provides an excellent opportunity for investigating the accuracy and reliability of different segmentation and classification algorithms for corneal ulcers, but also advances the development of new supervised learning based algorithms especially those in the deep learning framework.

## Abstract (summary)

**Table:** 

Measurement(s)	corneal ulcer
Technology Type(s)	staining
Factor Type(s)	ulcer severity • ulcer pattern
Sample Characteristic - Organism	Homo sapiens

Machine-accessible metadata file describing the reported data: 10.6084/m9.figshare.11371803

## Background & Summary

Corneal ulcer is the most frequently-occurred symptom in corneal diseases^1^. A corneal ulcer is an inflammatory or more seriously, infective condition of the cornea involving disruptions of its epithelial layer or the corneal stroma^2,3^. Corneal ulcers may be the consequences of contact lens, trauma, adnexal diseases, topical steroid uses, severe debilitation and ocular surface disorders and may result in corneal scarring, perforation, endophthalmitis, and decreased vision. Late or inappropriate treatment may induce irreversible damages to the human eyes^4,5^. Corneal ulcers have been significantly affecting the human eye health.

Fluorescein is the most widely used diagnostic dye in optometry and ophthalmology for assessing the integrity of the ocular surface and particularly the integrity of the cornea^6,7^. In many ophthalmologic examinations, corneal staining is one of the most commonly used methods for diagnosing corneal ulcers. Advances in staining techniques have allowed researchers to diagnose and analyze corneal ulcers together with a slitlamp microscopy. More importantly, the fluorescein staining imaging technique provides a great opportunity for quantitative analyses of corneal ulcers.

To quantify the severity of a corneal ulcer, its segmentation is desired, especially for flaky ulcers. Manual segmentation is considered as being the most reliable but highly time-consuming and of large inter- and intra-rater variability. During the past decade, various semi-^8–10^ and fully-automated^11–14^ techniques have been developed for segmenting corneal ulcers. Many of these segmentation algorithms employ machine learning techniques which require large training datasets. A training sample for image segmentation has two components: (1) the image used for segmentation; (2) the associated ground truth segmentation label. The size and diversity of the training dataset usually have significant influences on the performance of a machine learning based segmentation algorithm^8,15^. In the field of medical image segmentation, developing high-quality and large training datasets has become a very important research topic. For example, the LPBA40 dataset was created for the development of automated segmentation algorithms of 56 brain regions from structural magnetic resonance (MR) images^16,17^. A publicly accessible dataset on white matter hyper-intensity segmentation, from T1-weighted and FLAIR MR images, has been created and used in a MICCAI segmentation challenge^18^. The ATLAS dataset was established for developing automated algorithms for segmenting brain stroke lesions from structural MR images^19^. The IDRiD dataset was created to incite automated lesion detection algorithms from color fundus images with signs of diabetic retinopathy^20^. And the DRIVE database was established to advance automated segmentation algorithms of blood vessels from retinal images^21^. However, so far, there has been no publicly available training dataset that can be used for supervised learning based segmentations of corneal ulcers. In addition, to quantitatively compare the performance of different segmentation algorithms of corneal ulcers, it is of great importance to have an evaluation dataset with the ground truth segmentation labels being available.

In such context, we prepare a large corneal ulcer dataset, including ocular staining images and the corresponding segmentation labels of flaky corneal ulcers. In addition to flaky segmentation labels, we also provide each image with three-fold class labels: firstly, each image has a label in terms of its general ulcer pattern; secondly, each image has a label in terms of its specific ulcer pattern; thirdly, each image has a label indicating its ulcer severity degree. In terms of the general ulcer pattern, this dataset includes three categories respectively corresponding to point-like corneal ulcers, point-flaky mixed corneal ulcers and flaky corneal ulcers. Please note, the ground truth segmentation labels are exclusive to point-flaky and flaky corneal ulcers but not for point-like ones. In terms of the specific ulcer pattern and the severity degree, this dataset can be divided into both five categories respectively corresponding to five specific types of ulcer patterns and five grades of ulcer severity according to specific type-and-grading (TG) classification standards^22^. As such, this dataset is not only useful for developing and evaluating automated corneal ulcer segmentation algorithms but also beneficial for inventing and validating classification algorithms for identifying the general and specific ulcer patterns as well as the ulcer severity degree. A TG identification of a corneal ulcer utilizing a fluorescein staining image plays an important role in establishing individualized drug or surgical intervention strategies. It is a premise and basis for precisely treating various ocular surface diseases. This dataset can also be used as objective and quantitative indicators for evaluating different types of treatments and tracking their efficacy.

## Methods

### Data collection

A total of 712 fluorescein staining images capturing the ocular surfaces were collected from patients of various corneal ulcer degrees at the Zhongshan Ophthalmic Center at Sun Yat-sen University. There were no selection criteria on age, sex, ulcer severity, nor ulcer cause. Informed consent was obtained from each patient. The study protocol was approved by a properly constituted institutional review board (Zhongshan Ophthalmic Centre ethics committee of Sun Yat-sen University, Guangzhou, China), and the study was conducted in accordance with the ethical principles of the Declaration of Helsinki (2017KYPJ104).

The specific process of obtaining each fluorescein staining image is as follows: Staining was evaluated after a fluorescein-impregnated strip got wet with a single drop of sterile saline, and any excessive fluid was shaken off when the drop saturated the impregnated tip of both eyes of each patient. And then, the patient was asked to blink several times to ensure proper mixing of the fluorescein dye throughout the tear film. No rinsing step was used. Because the residence time is known to influence fluorescence, all images were taken 3–5 minutes after fluorescein instillation. At the slit lamp, clinical grading of the staining was conducted for all corneal regions. For the purpose of this study, we focused on the cornea area but not the conjunctival areas, given that the cornea area is the region that is most commonly and highly stained in corneal epithelial lesions.

We used slit-beam lighting with the maximum width (30 mm) of the white light source, a blue excitation filter, a 10 or 16 magnification, and a diffusion lens at a 10 to 308 oblique angle, with the light source sitting at the midpoint between the pupil margin and limbus. An automated digital camera system was used to set the aperture, exposure time, and shutter speed based on the luminance of the examination room. The images were obtained using a Haag-Streit BM 900 slit-lamp microscope (Haag Streit AG, Bern, Switzerland) in combination with a Canon EOS 20D digital camera (Canon, Tokyo, Japan). Images were saved in JPG format in 24-bit RGB color with a resolution of 2592 × 1728 pixels. Each image contained only one cornea, which was fully presented in the image and approximately centred in the visual field.

### Image Categorization

#### Three categories in terms of each ulcer’s general pattern

According to the shape and distribution characteristics of the corneal ulcers, they can be classified into three categories respectively corresponding to point-like corneal ulcers, point-flaky mixed corneal ulcers and flaky corneal ulcers. A point-like corneal ulcer indicates the presence of a corneal disease that is the most common and relatively mild. Point-like corneal ulcers typically manifest at an early stage of an ophthalmic inflammation or when the corneal ulcers are almost cured. This type of corneal ulcers usually has the pattern of a concentration of numerous small ulcer dots and can be distributed anywhere within the cornea, and they will typically recover after appropriate and effective treatments^23^. A flaky corneal ulcer indicates the presence of a most serious corneal disease, and the ulcer area generally has bright green color with clear boundaries. A flaky corneal ulcer may induce scars of varying thicknesses on the ocular surface, which will significantly affect the patient’s vision, and may even induce a loss of vision^24^. A point-flaky mixed corneal ulcer is irregularly distributed, containing both point-like and flaky ulcers within the cornea. A point-flaky mixed corneal ulcer usually indicates the presence of a corneal disease the severity degree of which lies between the aforementioned two types.

**Fig. 1:**
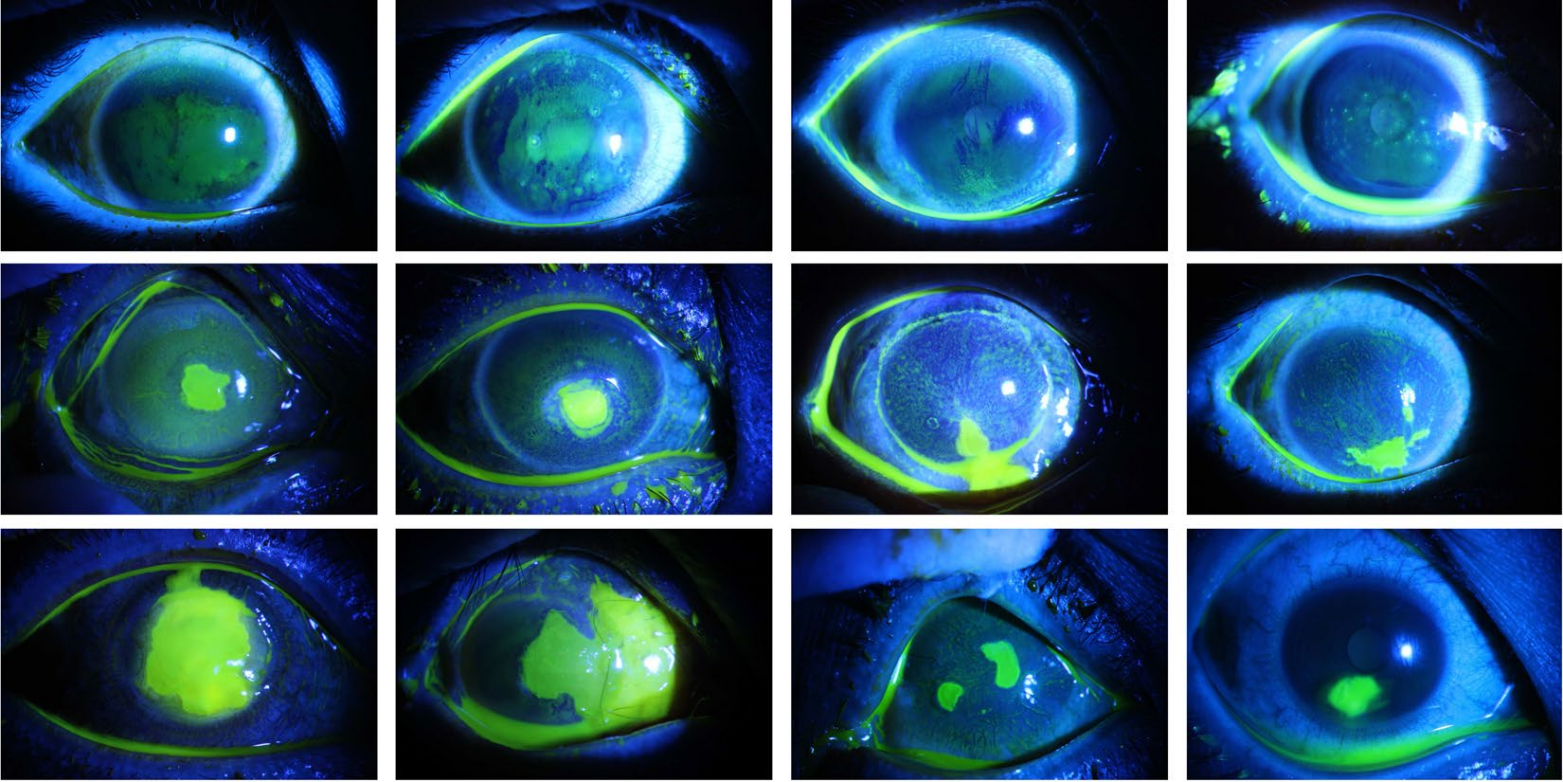
Demonstration of the three types of corneal ulcers in terms of their general patterns, with the top row representing point-like corneal ulcers, the middle row representing point-flaky mixed corneal ulcers and the bottom row representing flaky corneal ulcers.

Clinically, doctors or physicians need to adopt different treating and curring strategies for the aforementioned three categories of corneal ulcers. Therefore, correctly discriminating those three categories is of great clinical importance. Three experienced ophthalmologists from the Zhongshan Ophthalmic Centre at Sun Yat-sen University, who were responsible for ophthalmic research and clinical work, classified each image into the aforementioned three categories. Two ophthalmologists independently classified each ocular staining image, and a third ophthalmologist re-classified the images with inconsistent classification results from the previous two ophthalmologists. The entire dataset was distributed as follows: 358 images were labeled as having point-like corneal ulcers, 263 images were labeled as having point-flaky mixed corneal ulcers and the other 91 were labeled as having flaky corneal ulcers. Representative cases of those three types are demonstrated in Fig. 1.

### TG grading categories

The TG grading is a common staining grading method that has been worldwidely used. The TG grading method has two components: one is to classify each image according to the corneal ulcer’s specific pattern (Type grading), and the other is to classify each image according to the corneal ulcer’s severity degree (Grade grading)^22^. These two grading criteria are also important bases for a determination of subsequent treating plans.

**Fig. 2:**
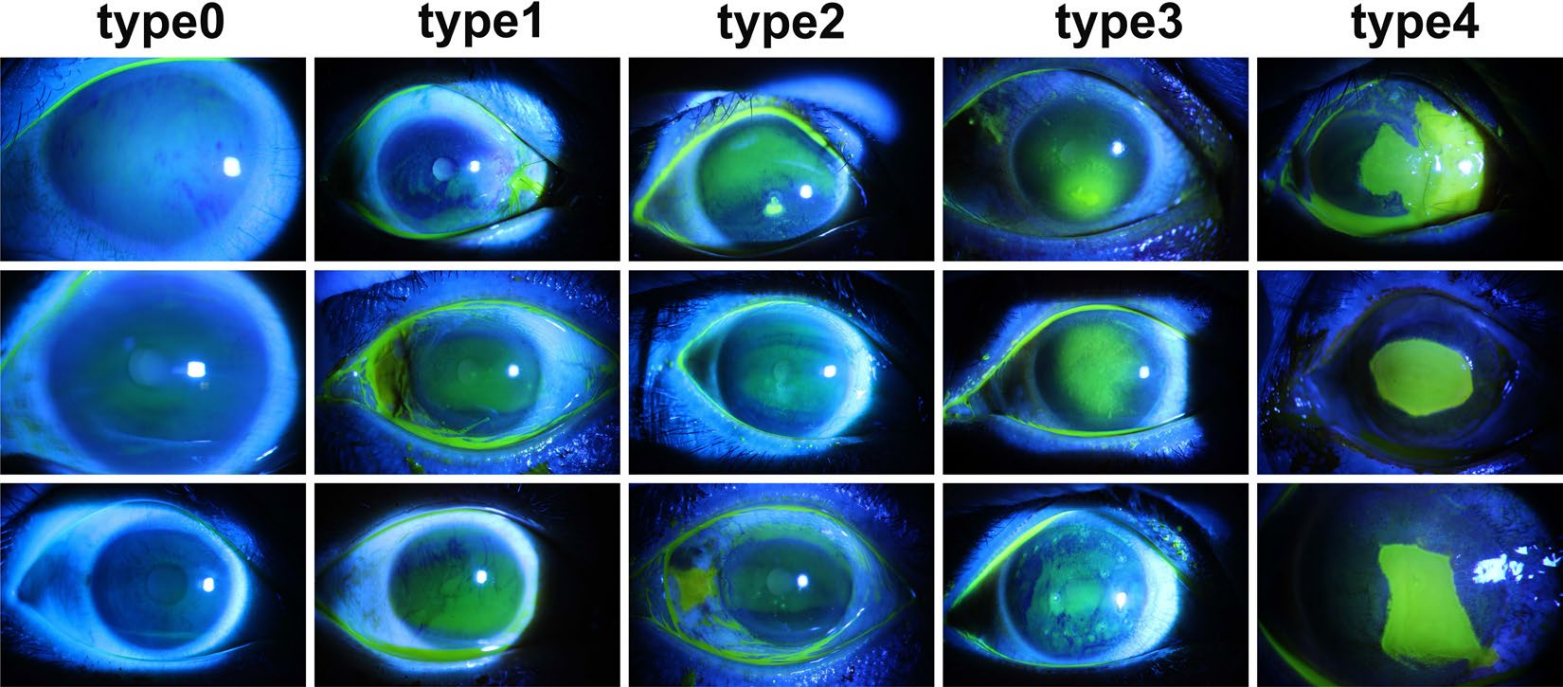
Demonstration of representative images corresponding to each of the five types in TG grading.

**(1) Type grading:** There are different types of corneal ulcers in terms of their specific patterns, and a judgment of the specific type is usually the first step for doctors to diagnose the potential underlying disease. The specific type also has an important impact on the corresponding treatment plan. We divided all images into 5 categories (type0–type4) according to the Type grading criteria^22^, as shown in Fig. 2. To be specific,

• type0: No ulcer of the corneal epithelium;

• type1: Micro punctate;

• type2: Macro punctate;

• type3: Coalescent macro punctate;

• type4: Patch (>=1 mm).

**(2) Grade grading:** The severity degree of a corneal ulcer is closely related to its location in the cornea. The pupil has a small circle shape located at the center of the cornea, which is the passage of light into the eye^25^. There are significant influences on vision when epithelium at the pupil is damaged. When ulcer occurs at the pupil area, the ulcer secretion material directly blocks the entry of light and affects vision. There may be situations where ulcers spread during a treatment, which may indicate that the corneal infection is aggravated. And it suggests that the current medical treatment cannot inhibit the growth of microorganisms and the treatment plan should be changed immediately. The position information of a corneal ulcer provides a reference for doctors to make treatment decisions.

**Fig. 3:**
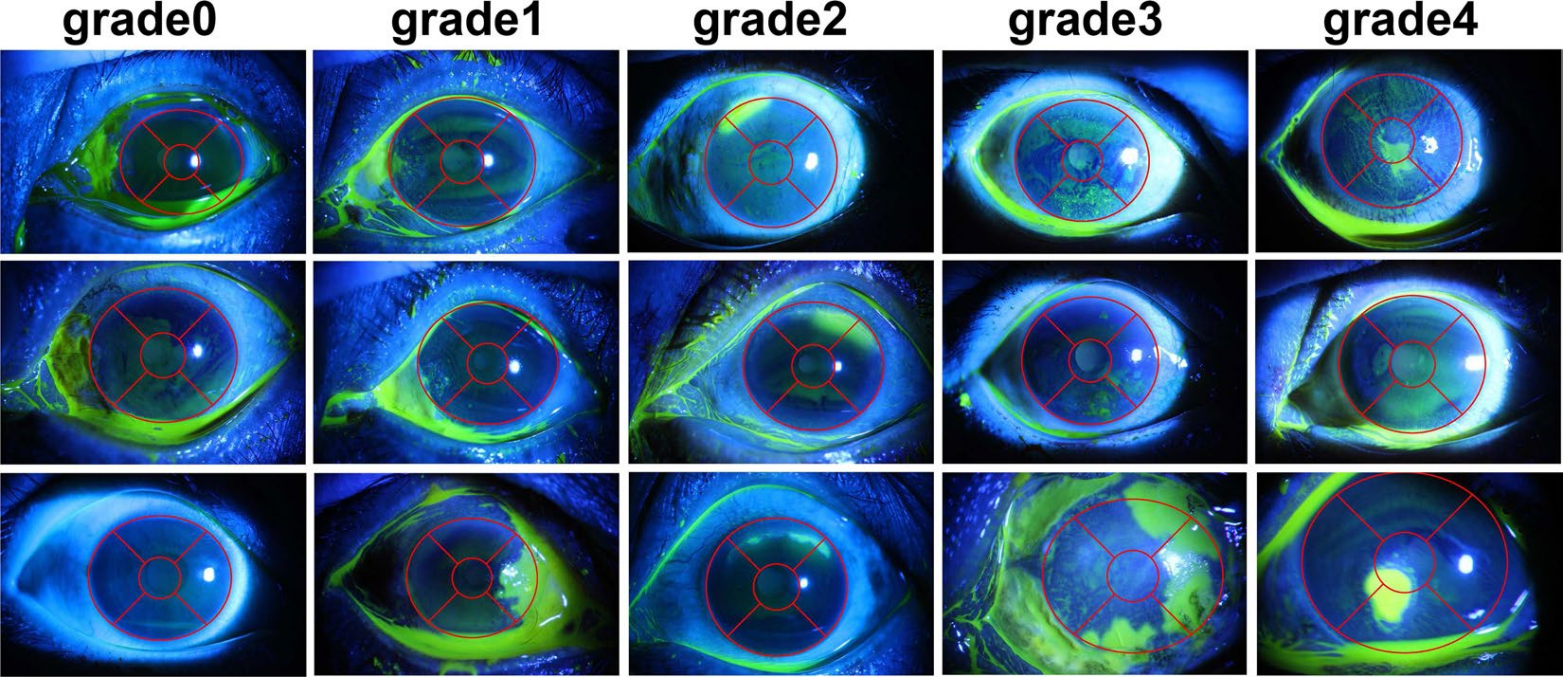
Demonstration of representative images corresponding to each of the five grades in TG grading.

The corneal surface can be divided into five quadrants based on our previously-proposed elliptical model used for corneal extraction^10^. The area where the pupil is located at is set to be the first quadrant, which is called the central optical zone of the cornea. The central optical zone is a circle located at the center of the cornea, and its radius is usually one-third of the axis of the ellipse contouring the cornea. The remaining four quadrants are evenly distributed in the ring area in a 90° fan shape^22^. Each image was graded according to the following criteria^22^, as shown in Fig. 3. To be specific,

• grade0: No ulcer of the corneal epithelium;

• grade1: Corneal ulcers involve only one surrounding quadrant;

• grade2: Corneal ulcers involve two surrounding quadrants;

• grade3: Corneal ulcers involve three or four surrounding quadrants;

• grade4: Corneal ulcers involve the central optical zone of the cornea.

Overall, most of our ocular staining images lie in the categories of type2 and type3. The specific distributions are: 36 images for type0, 98 images for type1, 203 images for type2, 273 image for type3, and 102 images for type4. In terms of the five grades, more than half of our ocular staining images lie in the category of grade5. The specific distributions are: 36 images for grade0, 78 images for grade1, 40 images for grade2, 10 image for grade3, and 548 images for grade4. Table 1 tabulates the numbers of images for each category in the Type grading and the Grade grading.

**Table 1:** The number of images belonging to each category in the TG grading.

Type grading	type0	type1	type2	type3	type4
Number of images	36	98	203	273	102
**Grade grading**	**grade0**	**grade1**	**grade2**	**grade3**	**grade4**
Number of images	36	78	40	10	548

### Creation of flaky ulcer segmentation labels

**Fig. 4:**
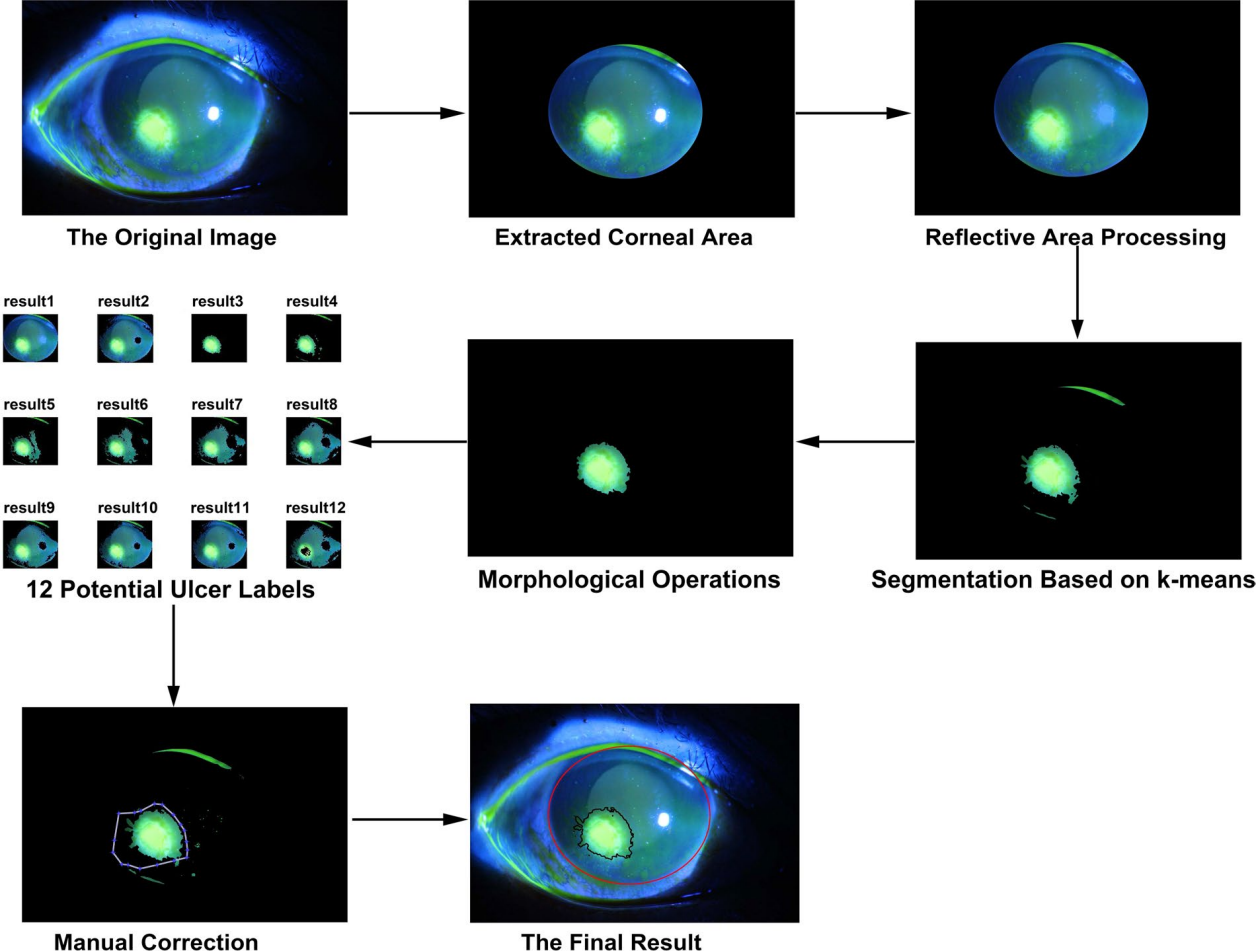
Demonstration of the flowchart of creating the ground truth segmentation labels of flaky corneal ulcers. The red outline in “The Final Result” indicates the boundary of the cornea, and the black outline indicates the boundary of the corneal ulcer.

For point-like corneal ulcers, it is very challenging to manually delineate the ulcer points. As such, we only prepare the ground truth segmentation labels for flaky ulcers in both flaky ulcer images and point-flaky mixed ulcer images. We first used an existing semi-automatic approach to identify the cornea from each image^10^. The main ulcer area was then segmented within the cornea by employing a combination of techniques: 1) identify and modify the color information of reflective areas in the original fluorescein staining image of interest; 2) iterative k-means based clustering to extract areas with similar color information; 3) morphological operations to polish results from the previous step, with the parameters employed in the morphological operators determined automatically via linear regression analyses. Detailed information of each step can be found in our previous works^10,26^. After extracting the main ulcer area, to provide more candidates for the subsequent manual correction, we created another 11 potential ulcer labels, as shown in Fig. 4.

In Fig. 4, result1 denotes the cornea with the reflective area having been processed. Result2 was obtained by removing specific pixels from result1. To be specific, pixels having the following RGB intensity ranges: 52 < R < 255, 37 < G < 255, 240 < B < 255 (approaching white), 150 < R < 255, 150 < G < 255, 150 < B < 255 (approaching pure blue), or 0 < R < 255, 0 < G < 10, 0 < B < 10 (approaching red) have been removed from result1 to yield result2. Those thresholding upper and lower bounds were identified based on empirical experiments. Result3 denotes the aforementioned segmentation result of the main ulcer area. After that, we removed result3 from result2 followed by a RGB-to-HSV conversion in terms of the pixel-wise color intensities^27^. We then calculated the average value of the H-channel and used it as an equal-point to divide all pixels into two piles. Each pile of pixels was then divided into another two piles in the same way until all pixels have been divided into eight non-overlapping piles. The average values of the H-channel of these eight piles had an monotonic increasing pattern. The pixels of these eight piles were sequentially added to result3 to respectively form the potential ulcer labels from result4 to result11. The reason why we created eight thresholding ranges is because that corneal ulcers typically have very different intensity ranges, varying from image to image. A single threshold would not work for all images. Result12 denotes the result after removing the pixels with intensity values lying in the range of 0 < H < 0.544, 0 < S < 1, 0 < V < 1 from result2^27^.

From the 12 potential ulcer labels, we manually selected the most accurate one, the one with the most complete ulcer area and the least background area. Please note, the result may still be not sufficiently accurate even after going through all aforementioned steps. For certain cases, based on ophthalmologists’ visual examinations, we performed manual corrections to obtain the final segmentation results of the flaky corneal ulcer labels. All the aforementioned steps are illustrated in Fig. 4.

## Data Records

This dataset is publicly available at (https://github.com/CRazorback/The-SUSTech-SYSU-dataset-for-automatically-segmenting-and-classifying-corneal-ulcers and 10.6084/m9.figshare.c.4526675), which can be downloaded as a zip file^28^. In this zip folder, all the original ocular staining images, the associated cornea labels, and the associated flaky ulcer segmentation labels (images with point-like corneal ulcers do not have such segmentation labels) are provided as three folders, named respectively as “rawImages”, “corneaLabels”, and “ulcerLabels”. Results of superimposing cornea and flaky corneal ulcer segmentation masks on the associated ocular staining images are also provided in the “corneaOverlay” and “ulcerOverlay” folders. In the “rawImages” folder, files are named as “n.jpg”, with n ranging between 1 and 712 denoting the nth sample. All images in this folder are saved in the JPG format. The segmentation label images of the cornea and the flaky ulcer area are saved as binary images in the PNG format, having the same size as the corresponding ocular staining image. An excel file was also provided, with the first column denoting the sample ID, the second column denoting the general pattern category (0 to 2, with 0 representing a point-like corneal ulcer, 1 representing a point-flaky mixed corneal ulcer, and 2 representing a flaky corneal ulcer), the third column denoting the type grading (0 to 4), and the fourth column denoting the grade grading (0 to 4). Two videos respectively demonstrating the semi-automatic segmentation process and the subsequent manual correction process were also provided in the zip folder.

## Technical Validation

All the segmentation labels of the 354 images with flaky corneal ulcers (263 images with point-flaky mixed corneal ulcers and 91 images with flaky corneal ulcers) have been visually reviewed independently by two ophthalmologists to ensure accuracy. It is worth being pointed out that among all those 354 images there are 100 images the flaky ulcer segmentation labels of which were created in a fully-manual way. Those 100 images had already been used in one of our previous works^26^.

The quality of all the 712 fluorescein staining images used in this study has been visually examined by experienced ophthalmologists to ensure sufficient image quality. A potential limitation of this work is that we did not provide any quantitative metrics to validate the quality of each image. A viable approach is to manually score the quality of each image, as proposed elsewhere^29^, which may also inaugurate deep learning approaches for automatically scoring the quality of fluorescein staining images.

The segmentation label of each image was provided in a binary format, even though the consensus was arrived by 3 independent annotators. A potential better way is to provide a probabilistic segmentation label, similar to what has been done for the DiaretDB1 dataset^30^. Recently, uncertainty has been paid great attention to in medical related deep learning tasks. There are two major types of uncertainty. Aleatoric uncertainty accounts for noises in the data, often induced during the data acquisition process. Epistemic uncertainty relates to uncertainty in the model parameters, usually arising from the limited size of datasets^31^. In this study, our sophisticated data acquisition procedure has reduced the aleatoric uncertainty to a relatively low extent, as verified by visual assessments. Multiple annotations in a probabilistic setting may help calibrate the estimation of aleatoric uncertainty^32^, which will be one of our future endeavors.

The dataset provided in this work has a relatively large sample size in terms of medical image segmentation tasks (a total of 354 samples), as compared to a sample size of 81 in the IDRiD dataset^20^ and a sample size of 40 in the DRIVE dataset^21^. However, in terms of TG classifications, this dataset is largely unbalanced and has a relatively limited sample size for certain categories, which may cause overfitting problems if used in a deep learning setting. In this context, this dataset may better suit “small dataset” regimes in terms of developing automated classification algorithms, the research topic of which has already been extensively investigated^33^. One of our future research efforts is to collect more data to address this limitation.

## Usage Notes

The dataset presented in this paper can be downloaded through the link mentioned before. Users of this dataset should acknowledge the contributions of the original authors and properly cite this article.
